# TRAP1 Regulates Wnt/β-Catenin Pathway through LRP5/6 Receptors Expression Modulation

**DOI:** 10.3390/ijms21207526

**Published:** 2020-10-13

**Authors:** Giacomo Lettini, Valentina Condelli, Michele Pietrafesa, Fabiana Crispo, Pietro Zoppoli, Francesca Maddalena, Ilaria Laurenzana, Alessandro Sgambato, Franca Esposito, Matteo Landriscina

**Affiliations:** 1 Laboratory of Pre-Clinical and Translational Research, IRCCS, Referral Cancer Center of Basilicata, 85028 Rionero in Vulture, PZ, Italy; lettini.giacomo@gmail.com (G.L.); valentina.condelli@crob.it (V.C.); michele.pietrafesa@crob.it (M.P.); fabiana.crispo@crob.it (F.C.); pietro.zoppoli@crob.it (P.Z.); francescamaddalena77@gmail.com (F.M.); ilaria.laurenzana@crob.it (I.L.); alessandro.sgambato@crob.it (A.S.); 2 Department of Molecular Medicine and Medical Biotechnology, University of Naples Federico II, 80131 Naples, Italy; 3 Medical Oncology Unit, Department of Medical and Surgical Sciences, University of Foggia, 71100 Foggia, Italy

**Keywords:** molecular chaperone, TRAP1, Wnt signaling, cancer stem cell, colon cancer, stemness

## Abstract

Wnt/β-Catenin signaling is involved in embryonic development, regeneration, and cellular differentiation and is responsible for cancer stemness maintenance. The HSP90 molecular chaperone TRAP1 is upregulated in 60–70% of human colorectal carcinomas (CRCs) and favors stem cells maintenance, modulating the Wnt/β-Catenin pathway and preventing β-Catenin phosphorylation/degradation. The role of TRAP1 in the regulation of Wnt/β-Catenin signaling was further investigated in human CRC cell lines, patient-derived spheroids, and CRC specimens. TRAP1 relevance in the activation of Wnt/β-Catenin signaling was highlighted by a TCF/LEF Cignal Reporter Assay in Wnt-off HEK293T and CRC HCT116 cell lines. Of note, this regulation occurs through the modulation of Wnt ligand receptors LRP5 and LRP6 that are both downregulated in TRAP1-silenced cell lines. However, while LRP5 mRNA is significantly downregulated upon TRAP1 silencing, LRP6 mRNA is unchanged, suggesting independent mechanisms of regulation by TRAP1. Indeed, LRP5 is regulated upon promoter methylation in CRC cell lines and human CRCs, whereas LRP6 is controlled at post-translational level by protein ubiquitination/degradation. Consistently, human CRCs with high TRAP1 expression are characterized by the co-upregulation of active β-Catenin, LRP5 and LRP6. Altogether, these data suggest that Wnt/β-Catenin signaling is modulated at multiple levels by TRAP1.

## 1. Introduction

Wnt pathway is an evolutionarily conserved signaling broadly involved in regulating embryonic development, regeneration, and cellular differentiation [1]. Indeed, Wnt pathway consists of a large family of secreted glycoproteins responsible for a wide range of cellular functions, including cellular proliferation and migration, cells fate, and stem cell maintenance [2]. 

Wnt signaling is classified into canonical and non-canonical pathways, based on the dependency of its activity on β-Catenin. The canonical pathway (β-Catenin-dependent) controls self-renewal/differentiation and cellular proliferation [3], whereas the non-canonical pathway (β-Catenin-independent) is responsible for cell movement regulation during morphogenesis and inhibition of the canonical pathway [4].

In the canonical pathway, Wnt proteins act as ligands, interacting with two major classes of cell membrane receptors: Frizzled (FZD) and Lipoprotein-related proteins 5/6 (LRP5/6) [2]. Upon Wnt interaction with receptors, the intracellular signal is transduced with the recruitment of cytoplasmic Disheveled phosphoproteins (Dvl) to membranes and this provides a site for Axis Inhibitor (AXIN) and Glycogen Synthase Kinase-3 (GSK3β) to bind and phosphorylate LRP5/6, thus preventing the constitutive degradation of β-Catenin [5]. Intracellular Wnt/β-Catenin signaling depends on the amount of the transcriptional co-activator β-Catenin, which migrates from the cytoplasm to the nucleus to engage the T cell Transcription Factor (TCF) or the Lymphoid Enhancer Factor (LEF) regulating the expression of several target genes involved in key cellular processes, such as *c-Myc*, *cyclin D1*, and *CD44* [6,7]. In the absence of Wnt ligands, β-Catenin is degraded through phosphorylation and ubiquitination by a multimeric protein complex, known as destruction complex, consisting of several proteins, as Adenomatous Polyposis Coli (APC), AXIN, Casein Kinase 1 (CK1), and GSK3β [8].

Given its relevance in cellular homeostasis, the aberrant activation of Wnt pathway is a key mechanism in human colorectal carcinogenesis [7]. Aberrant mutations in Wnt pathway components have been identified in about 90% of human CRCs, especially inactivating APC mutations or activating β-Catenin mutations, resulting in enhanced transcription of genes involved in cellular proliferation and tumor progression [9]. In this contest, our group previously reported that TRAP1, a HSP90 molecular chaperone responsible for several key functions in human carcinogenesis [10,11,12,13,14] and upregulated in about 60–70% of human CRCs [15,16,17], favors stemness maintenance in CRC cells modulating Wnt/β-Catenin pathway [18]. Interestingly, TRAP1 is preferentially expressed by colorectal cancer stem cells (CSCs), enhances β-Catenin expression levels, and is co-expressed with several Wnt target genes in human CRCs [18]. Mechanistically, TRAP1 prevents the phosphorylation/ubiquitination of β-Catenin favoring the activation of Wnt signaling and this regulation occurs upstream to the degradation complex [18]. Based on this background, this study further highlights the role of TRAP1 in the regulation of Wnt/β-Catenin signaling, showing that it occurs through the control of LRP5/6 expression.

## 2. Results

### 2.1. TRAP1 Modulates the Activity of Wnt/β-Catenin Pathway

**Figure 1:**
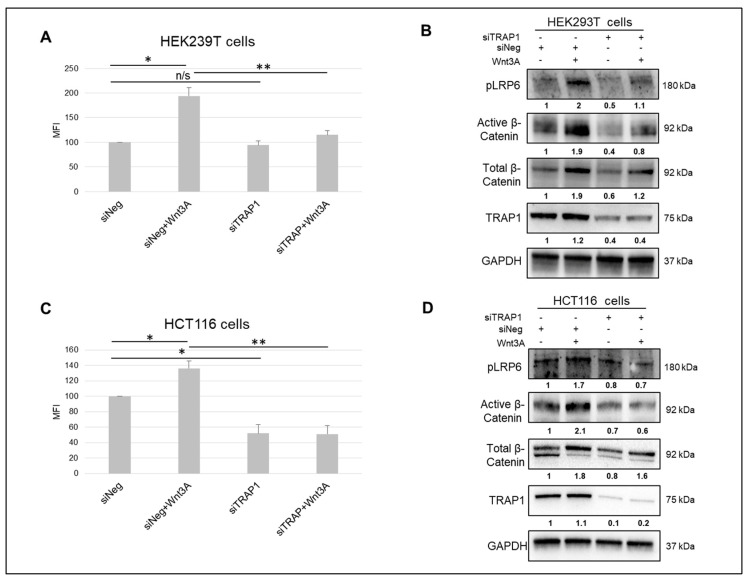
TRAP1 modulates the activity of Wnt/β-Catenin pathway. (**A**,**C**) The activity of Wnt/β-Catenin signaling was evaluated in control (siNeg) and TRAP1-silenced (siTRAP1) HEK293T (A) and HCT116 (C) cells, upon transfection with a Cignal TCF/LEF Reporter and subsequent stimulation with 150 ng/µL Wnt3A recombinant protein for 6 h. Statistical significance respect to the respective control: * *p* < 0.05; ** *p* < 0.01; n/s, not significant. Data represent means + S.D. MFI, Mean Fluorescence Intensity. (**B**,**D**) Total lysates from control and TRAP1-silenced HEK293T (B) and HCT116 (D) cells, treated as reported in panels (**A**,**C**), were separated by SDS-PAGE and immunoblotted with indicated antibodies.

We previously reported that TRAP1 favors stemness maintenance in CRC cells, and that this occurs through Wnt/β-Catenin pathway and the regulation of β-Catenin ubiquitination/phosphorylation process [18]. In order to further determine how TRAP1 modulates the activation of Wnt/β-Catenin signaling, we monitored the enhancing effect of Wnt3A ligand on the activity of Wnt/β-Catenin pathway in HEK293T cells (Figure 1A,B)—a Wnt OFF cell line carrying a functional destruction complex [19]—and in CRC HCT116 cells, which are characterized by a β-Catenin mutation [20] (Figure 1C,D).

This was achieved upon cell transfection with a Cignal TCF/LEF Reporter and subsequent stimulation with Wnt3A recombinant protein. Immunoblot analysis of active β-Catenin expression and Wnt receptor LRP6 phosphorylation were used as markers of pathway activation/inactivation. TRAP1 silencing resulted in the downregulation of total and active β-Catenin levels in both cell lines, as previously reported by Lettini et al. [18]. A significant induction of Wnt/β-Catenin pathway was observed in both cell lines upon Wnt3A stimulation, as showed by the increase in reporter gene expression (Figure 1A,C) and the parallel upregulation of active β-Catenin levels and LRP6 phosphorylation (Figure 1B,D). Intriguingly, TRAP1 silencing resulted in loss of pathway activation in response to Wnt3A in both cell lines (Figure 1A,C) and the downregulation of active β-Catenin levels and LRP6 phosphorylation (Figure 1B,D). The statistical analysis of densitometric data from three independent replicates of immunoblots showed in Figure 1B,D are reported in Figure S1. These data support the concept that TRAP1 is relevant for Wnt/β-Catenin pathway activation and that its downregulation results in Wnt signaling attenuation.

### 2.2. TRAP1 Enhances the Sphere-Forming Ability of Colon Carcinoma and Cancer Stem Cells 

**Figure 2:**
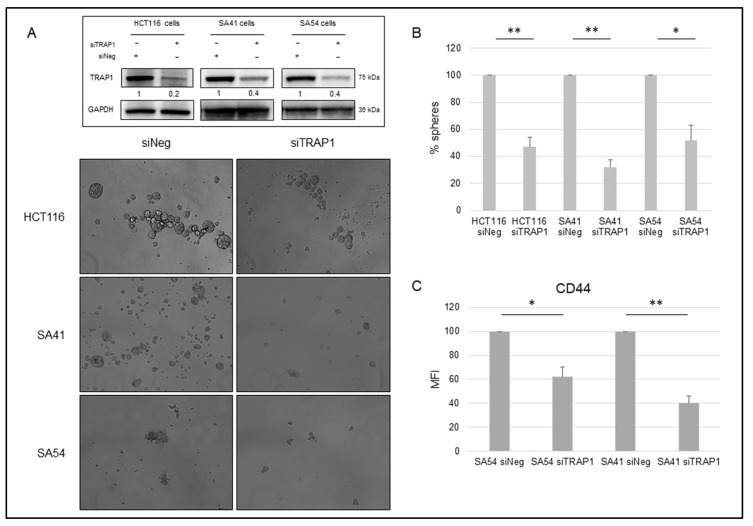
TRAP1 enhances the sphere-forming ability of colon carcinoma and cancer stem cells. (**A**,**B**) Representative images (**A**) and quantitative representation (**B**) of the sphere-forming ability in control (siNeg) and TRAP1-silenced (siTRAP1) HCT116, SA41 and SA54 cells. Statistical significance respect to the respective siNeg control: * *p* < 0.05; ** *p* < 0.01. Data represent means + S.D. (**A**) Insert: TRAP1 immunoblot analysis in siNeg and siTRAP1 HCT116, SA41 and SA54 cells. (**C**) Relative CD44 MFI levels in control (siNeg) and TRAP1-silenced (siTRAP1) SA41 and SA54 cells evaluated by CD44 immunostaining and subsequent flow cytometry. Statistical significance respect to the respective siNeg control: * *p* < 0.05; ** *p* < 0.01; n/s. Data represent means + S.D. MFI, Mean Fluorescence Intensity.

In subsequent experiments, we questioned whether TRAP1 modulates the spheroid-forming ability of CRC HCT116 cells and SA41 and SA54 patient-derived spheroids. TRAP1-silenced and relative parental control cells were seeded as single-cell suspensions and compared for their sphere-forming ability. Noteworthy, TRAP1 knocking down (Figure 2A, insert) resulted in a significant impairment of sphere formation (Figure 2A), with a 1:2 ratio in spheroid numbers between siTRAP1 and siNeg samples in HCT116 and SA54 cells, and a 1:3 ratio in SA41 cells (Figure 2B). Consistently, TRAP1 knocking-down induced a significantly downregulation of the stem marker and Wnt/β-Catenin target gene, CD44 in SA54 and SA41 spheroids (Figure 2C). These data suggest that TRAP1 is responsible for the sphere-forming ability and stemness maintenance in CRC CSC cells.

### 2.3. TRAP1 Regulation of Wnt/β-Catenin Pathway Activation Occurs through LRP5/6 Receptors

**Figure 3:**
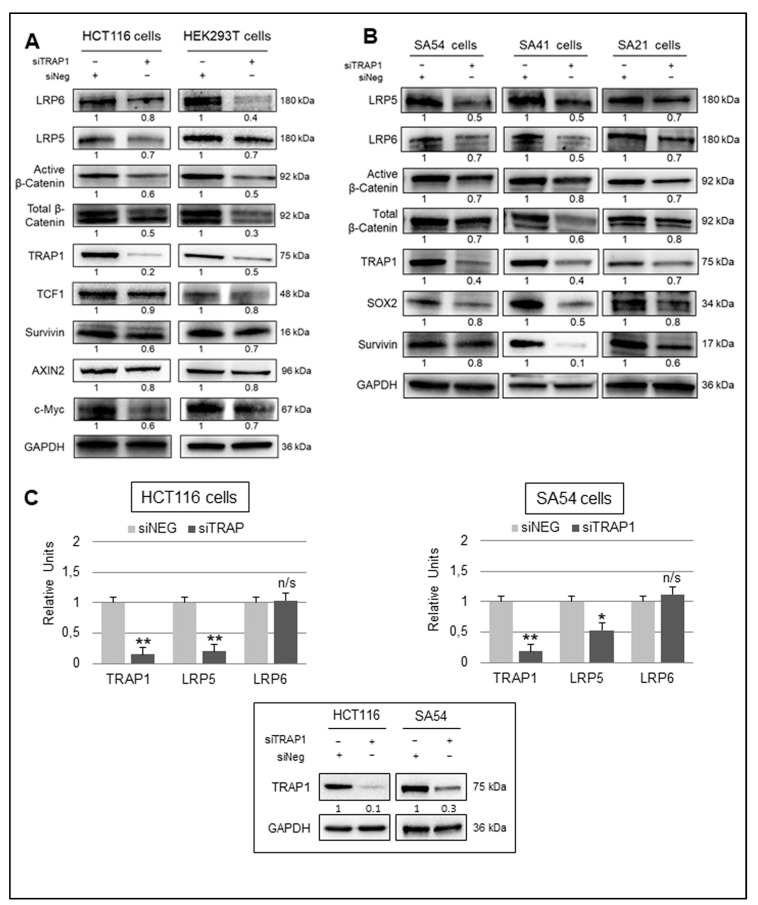
TRAP1 silencing results in the downregulation of LRP5/6 receptors and Wnt/β-Catenin-related proteins. (**A**,**B**) Total cell lysates collected from control (siNeg) and TRAP1-silenced (siTRAP1) HCT116 and HEK293T cells (**A**), and SA54, SA41, and SA21 patient-derived spheroids (**B**) were separated by SDS-PAGE and immunoblotted with the indicated antibodies. (**C**) Real-time PCR gene expression analysis of LRP5 and LRP6 in TRAP1-silenced HCT116 and SA54 cells compared to cells transfected with negative siRNA. Statistical significance respect to siNeg control: * *p* < 0.05; ** *p* < 0.01; n/s, not significant. Data represent means + S.D.

As TRAP1 modulates β-Catenin phosphorylation/degradation without being part of its degradation complex [18], the hypothesis that TRAP1 regulates upstream levels of Wnt canonical pathway activation was further investigated. More specifically, we studied the relationship between TRAP1 and Wnt LRP5/6 receptors, which are known to be widely expressed in colon epithelial and CRC cells [15,21,22]. The protein expression of LRP5/6 receptors and specific β-Catenin-dependent TCF target genes were evaluated in low versus high TRAP1 background in HCT116 and HEK293T cells (Figure 3A) and in patient-derived spheroids (Figure 3B) [14,18,23]. Indeed, TRAP1 silencing in HCT116 and HEK293T cells resulted in the downregulation of LRP5/6 receptors, and this was paralleled by the downregulation of active and total β-Catenin and several Wnt/β-Catenin target genes, as TCF1, Survivin, c-Myc, and Axin2 (Figure 3A). Similarly, TRAP1 downregulation impaired the expression of LRP5/6 receptors, active and total β-Catenin, Survivin, and the stem cell marker SOX2 in SA54, SA41, and SA21 spheroids (Figure 3B). The statistical analysis of densitometric data from three independent replicates of immunoblots shown in Figure 3A,B are reported in Figure S2. 

To prove the specificity of TRAP1 regulation on LRP5/6 receptors, the molecular chaperone was silenced in HCT116 cells by two independent siRNAs, and this resulted in a significant downregulation of LRP5 and LRP6 (Figure S3A). Consistently, the transfection of TRAP1 cDNA partially rescued the downregulation of LRP5/6 receptors in shTRAP1 HCT116 cells (Figure S3B). These data suggest that TRAP1 regulation of Wnt/β-Catenin pathway occurs upstream to β-Catenin destruction complex, likely on LRP5/6 receptors.

### 2.4. TRAP1 Regulates LRP5/6 Expression through a Double Distinct Molecular Mechanism

In order to further evaluate the molecular mechanism used by TRAP1 to modulate the expression of LRP5/LRP6 receptors, we questioned whether this regulation occurs at transcriptional level. Thus, mRNA levels of LRP5 and LRP6 were analyzed by RT PCR in CRC HCT116 cells and SA54 spheroids upon TRAP1 interference. LRP6 mRNA was unchanged after TRAP1 silencing in HCT116 and SA54 cells, whereas LRP5 mRNA was significantly downregulated (Figure 3C), even if the level of both proteins were impaired in TRAP1-silencing conditions. 

**Figure 4:**
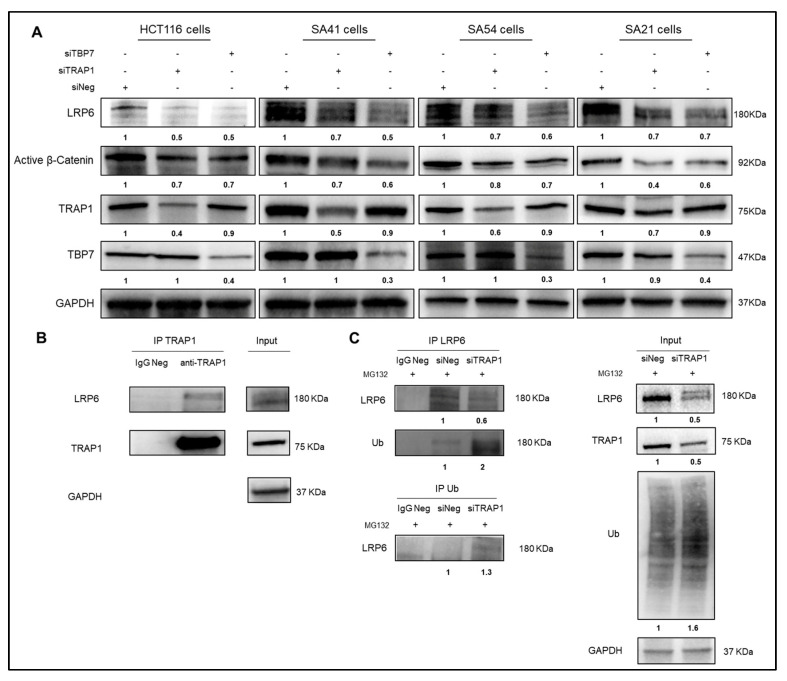
TRAP1 regulates LRP6 expression through a quality control by the regulation of its ubiquitination/degradation. (**A**) Total lysates from HCT116, SA41, SA54, and SA21 cells, transfected with control (siNeg) or TRAP1/TBP7 siRNAs, were separated by SDS-PAGE and immunoblotted with indicated antibodies. (**B**) Anti-TRAP1 immunoprecipitates were obtained from HCT116 cells, separated by SDS-PAGE and immunoblotted with anti-LRP6 and anti-TRAP1 antibodies. IgG Neg, total cellular extracts incubated with nonspecific antibody. Input: Total cell lysates from HCT116 cells were separated by SDS-PAGE and immunoblotted with the indicated antibodies. (**C**) Anti-LRP6 (upper panel) and anti-ubiquitin (lower panel) immunoprecipitates were obtained from HCT116 cells transfected with control (siNeg) or TRAP1 siRNAs, separated by SDS-PAGE and immunoblotted with anti-ubiquitin (upper panel) and anti-LRP6 (lower panel) antibodies. Cells were incubated with 10 mmol/L MG132 for 2 h before cell lysis. Densitometric band intensities represent the ratios between ubiquitinated and total LRP6 bands in IPs. IgG Neg, total cellular extracts incubated with nonspecific antibody. Input: Total cell lysates from HCT116 cells transfected with control or TRAP1 siRNA were separated by SDS-PAGE and immunoblotted with the indicated antibodies.

Thus, we hypothesized that TRAP1 regulation on LRP6 may occur at post-translational level. This hypothesis is consistent with the current knowledge about TRAP1 regulation of its protein network, as it is widely accepted that TRAP1 regulates the quality of its interactors on the endoplasmic reticulum, preventing their degradation [24]. This TRAP1 function is mediated by the cooperation with the proteasome regulatory particle TBP7, which prevents protein ubiquitination upon TRAP1 quality control [23,25]. Thus, we questioned whether LRP6 expression was impaired upon TBP7 interference, as observed under TRAP1 silencing conditions. Indeed, LRP6 and, consequently, active β-Catenin levels were downregulated upon TBP7 silencing as observed upon TRAP1 interference in HCT116, SA41, SA54, and SA21 cells (Figure 4A), suggesting that its expression is controlled by TRAP1/TBP7 network. Based on this evidence, we hypothesized that LRP6 is among TRAP1 interactors whose expression is regulated by ubiquitination/degradation.

In subsequent experiments, TRAP1 was immunoprecipitated from total lysates of HCT116 cells and TRAP1 immunoprecipitates were evaluated by LRP6 immunoblot analysis. Interestingly, a positive co-immunoprecipitation was observed between the two proteins (Figure 4B), suggesting a potential interaction. Furthermore, LRP6 was immunoprecipitated from HCT116 cells upon TRAP1 silencing and further assayed by anti-ubiquitin antibodies. Indeed, ubiquitin immunoblot analysis of LRP6 immunoprecipitates showed increase of its ubiquitination in TRAP1-interfered cells (Figure 4C, upper panel). In parallel, ubiquitinated proteins were immunoprecipitated from TRAP1-silenced HCT116 cells and further evaluated by anti-LRP6 antibodies to assess the level of ubiquitinated LRP6. Noteworthy, LRP6 immunoblot analysis of anti-ubiquitin immunoprecipitates showed increased levels of LRP6 in TRAP1-interfered cells despite the overall reduction of LRP6 levels (Figure 4C, input), and this is consistent with increase in overall protein ubiquitination in TRAP1-silenced cells (Figure 4C, input). These evidences suggest that LRP6 is regulated post-transcriptionally by TRAP1 by preventing protein degradation in HCT116 cells.

**Figure 5:**
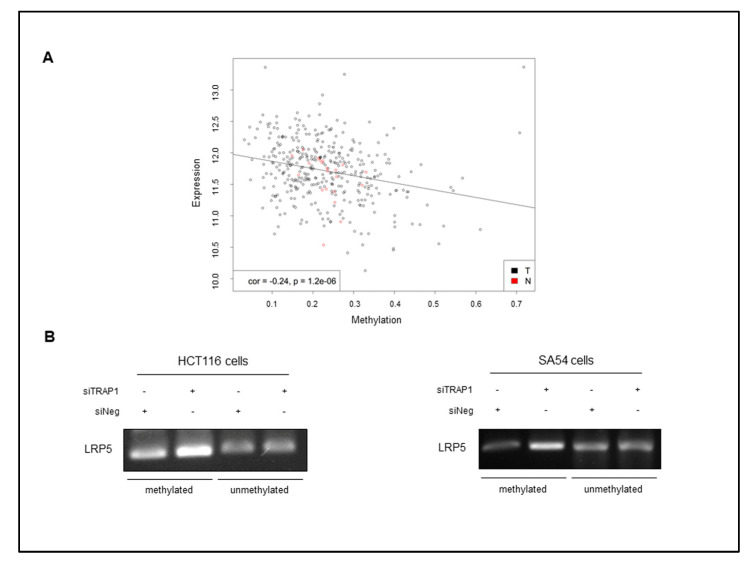
TRAP1 regulates LRP5 expression through DNA methylation mechanisms. (**A**) Scatter plot of 361 TCGA colorectal adenocarcinomas. Promoter methylation is reported on the *x*-axis, mRNA expression on the *y*-axis. Solid line shows the linear regression. Rho is the Spearman’s rank correlation and the *p*-value is the result of the correlation test. (**B**) Methylation-specific PCR representing the level of LRP5 promoter methylation in control (siNeg) TRAP1-silenced (siTRAP1) HCT116 and SA54 cells. PCR analysis of a LRP5 un-methylated region is reported as internal control.

As LRP5 mRNA level was significantly downregulated in parallel with its protein level, we questioned whether the downregulation of LRP5 was due to a transcriptional control by TRAP1 protein network. To address this issue, we took advantage from literature data, which suggest that LRP5 expression is controlled by DNA methylation mechanisms in in vitro cell models and in human malignancies [26,27]. Thus, the TCGA dataset was used to study the relationship between LRP5 promoter methylation and its mRNA expression in a cohort of 361 human CRCs. Interestingly, an inverse statistically significant correlation between LRP5 promoter methylation and mRNA levels was observed (Figure 5A), this supporting the relevance of epigenetic mechanism in LRP5 regulation in human CRC. Thus, to further validate this preliminary evidence, the methylation level of LPR5 promoter was evaluated in TRAP1-silenced HCT116 cells and SA54 spheroids by a PCR assay specifically designed to recognize methylated regions in LRP5 promoter. PCR analysis of a LRP5 unmethylated region was used as internal control. Intriguingly, LRP5 promoter methylation levels were increased after TRAP1 interference, whereas the LRP5 unmethylated region were unchanged in the same experimental conditions in both HCT116 and SA54 cell lines (Figure 5B). Altogether, these observations suggest that TRAP1 regulation of LRP5/LRP6 receptors occurs by a dual mechanism: LRP6 is among the proteins regulated at post-translational level by TRAP1/TBP7 quality control network, whereas LRP5 expression is regulated at transcriptional level by promoter methylation.

### 2.5. TRAP1 Is Co-Expressed with Active β-Catenin and LRP5/6 in Human CRCs

**Figure 6:**
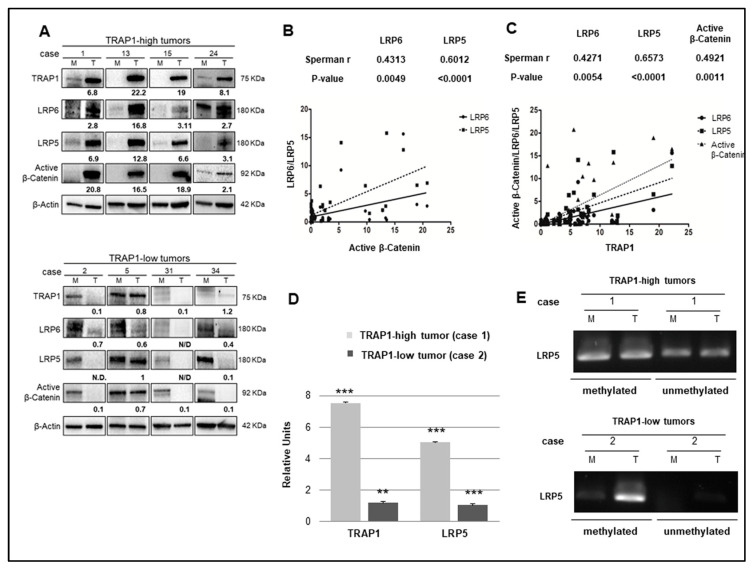
TRAP1 is co-expressed with active β-Catenin and LRP5/6 in human CRCs. (**A**) Total cell lysates from eight representative human CRCs (T) and the respective peritumoral non-infiltrated mucosas (M) were separated by SDS-PAGE and immunoblotted with the indicated antibodies. (**B**,**C**) Dot plots representing the correlation between active β-Catenin and LRP5/LRP6 (**B**) and between TRAP1 and active β-Catenin/LRP5/LRP6 (**C**) in our series of 41 colorectal carcinomas. (**D**) Real Time PCR analysis of LRP5 mRNA expression in two representative cases of human CRCs, represented as time increase compared to the respective non-infiltrated normal mucosa. Statistical significance respect to the respective control: ** *p* < 0.01; *** *p* < 0.001. (**E**) Methylation specific PCR representing the level of LRP5 promoter methylation in two representative cases of human CRCs.

To address the relevance of TRAP1 regulation on Wnt/β-Catenin pathway through LRP5/LRP6 in vivo, an in-house cohort of 41 human CRCs (Table 1) was evaluated for the expression of active β-Catenin, LRP5, and LRP6 and their levels were correlated with TRAP1 protein expression (Figure 6 and Table S1).

The Spearman Rank test was used to determine the statistical significance of the correlation between TRAP1 and active β-Catenin, LRP5, and LRP6 protein expression. Interestingly, a significant co-expression between TRAP1 and active β-Catenin, LRP5, and LRP6 proteins was observed, with marked upregulation of LRP5/6 and active β-Catenin in tumors with higher TRAP1 expression (Figure 6A). Intriguingly, in a preliminary analysis, the Spearman Rank test showed a statistically significant correlation between active β-Catenin levels and LRP5/LRP6 receptors (LRP6 Sperman r: 0.43, *p*-value: 0.005; LRP5 Sperman r: 0.6, *p*-value: <0.0001), confirming the dependence of active β-Catenin expression on Wnt LRP5/6 receptors (Figure 6B). Consistently, the Spearman Rank test exhibited a statistically significant correlation between TRAP1 and LRP5, LRP6, and active β-Catenin in the same cohort of human CRCs (LRP6 Sperman r: 0.43, *p*-value: 0.005; LRP5 Sperman r: 0.66, *p*-value: <0.0001; active β-Catenin Sperman r: 0.49, *p*-value: 0.001) (Figure 6C). These data support the hypothesis that TRAP1 regulation on Wnt/β-Catenin pathway occurs through the modulation of the expression of LRP5/6 receptors and that this mechanism is conserved in human CRCs.

Finally, to further support the role of epigenetic mechanisms in LPR5 regulation by TRAP1 in vivo, LRP5 mRNA expression and promoter methylation were assessed in a few CRC specimens, selected based on the availability of DNA, RNA and protein extracts from the same sample. Two representative cases are reported in Figure 6D,E. Interestingly, higher levels of LRP5 promoter methylation and lower mRNA expression were observed in human CRCs characterized by low TRAP1 expression compared to high-TRAP1 tumors (Figure 6D,E).

## 3. Discussion

Several molecular chaperones are determinants of cancer cell stemness and potential targets for therapeutic approaches directed against CSCs [28,29]. They are involved in the maintenance of the CSC phenotype and are components of the cytoprotective machinery, allowing CSCs to survive to unfavorable environments. In such a context, the HSP90 molecular chaperone, TRAP1 is upregulated in the majority of human CRCs [15,30] and is preferentially expressed by intestine stem cells and CSCs isolated from CRC cell lines [18]. Noteworthy, TRAP1 enhances the anchorage-independent growth of CRC cells and the sphere-forming ability of CRC CSCs (see the work in [18] and this manuscript) and its knocking down induces the loss of the stem-like signature with gain of a more differentiated phenotype [18]. Moreover, several features of TRAP1 biology and its role in cancer cells are consistent with its function in stemness maintenance. TRAP1 represents a key determinant of cancer cell adaptation to different environmental conditions (i.e., oxidative, endoplasmic reticulum, and metabolic stress), being responsible for protection toward apoptosis, mitochondrial integrity, and drug resistance [31,32,33]. TRAP1 is also responsible for reprogramming of tumor bioenergetics as it downregulates oxidative phosphorylation, upon inhibition of succinate dehydrogenase, and enhances Warburg metabolism [34,35]. Thus, TRAP1 upregulation is a valuable strategy to protect CSCs from apoptotic cell death and adapt their metabolism to the hypoxic niche environment [18] and likely provides a potential molecular target for CSC-directed therapeutic strategies.

TRAP1 regulation of CSCs occurs through the modulation of Wnt/β-Catenin signaling, by preventing β-Catenin phosphorylation and degradation [18]. However, while TRAP1 interacts with β-Catenin, without being a component of β-Catenin destruction complex, its regulation likely occurs upstream to β-Catenin degradation complex [18]. Here, we report that TRAP1 enhances the expression of Wnt receptors, LRP5 and LRP6 thus favoring the activation of Wnt/β-Catenin signaling. Indeed, both LRP5 and LRP6 receptors are downregulated upon TRAP1 interference and this correlates with the inhibition of Wnt/β-Catenin pathway and loss of sphere formation in patients-derived CRC CSCs. Moreover, a statistically significant co-expression was observed between TRAP1, active β-Catenin, and LRP5/LRP6 receptors in human CRCs. In such a context, it is widely accepted that LRP5/LRP6 receptors occupancy by Wnt ligand induces a cascade of events resulting in inhibition of the destruction complex, prevention of β-Catenin phosphorylation and activation of Wnt-responsive genes [6]. Thus, it is reasonable to hypothesize that TRAP1 regulation of LRP5 and LRP6 expression is among the mechanisms responsible for the activation of Wnt/β-Catenin signaling, preventing β-Catenin phosphorylation/degradation.

The mechanism of TRAP1 regulation of LRP5 and LRP6 receptors is quite complex. LRP6 is controlled at post-translational level by preventing its ubiquitination and degradation. Indeed, LRP6 interacts with TRAP1 and its ubiquitination is enhanced in conditions of low TRAP1 expression. This observation is consistent with TRAP1 role in proteostasis, as this molecular chaperone plays a co-translational quality control on selective client proteins, most of them key genes involved in cancer progression [16]. This regulation occurs at the interface between the endoplasmic reticulum and the cytosol by the cooperation with the proteasome regulatory protein, TBP7, as observed also for LRP6. 

More complex is the mechanism of TRAP1 regulation of LRP5. Indeed, no interaction was observed between the TRAP1 and LRP5 and this correlated with lack of ubiquitination of LRP5 in TRAP1 silencing conditions (data not shown). Noteworthy, LRP5 transcript was downregulated upon TRAP1 knocking down, this suggesting a transcriptional control by TRAP1 protein network. Interestingly, our data suggest that LRP5 gene promoter is hypermethylated in condition of low TRAP1 expression in CRC cell lines, patient-derived spheroids and human CRC samples and this correlates with mRNA and protein downregulation. This observation is consistent with the evidence that LRP5 expression is regulated by DNA methylation mechanisms in in vitro cell models and in human malignancies [26,27] and with the evidence of an inverse correlation between LRP5 promoter methylation and its mRNA expression in a cohort of human CRCs from the TCGA database. While the evidence that TRAP1 protein network is responsible for reprogramming of DNA methylation events is a new finding, recent reports suggest a role for HSP90 as “epigenetic capacitor”. Indeed, HSP90 contributes to the phenotypic plasticity and modulates epigenetics by interacting with chromatin, chromatin regulators, and epigenetic effectors [36]. Furthermore, chromatin remodeling is at the crossroad between metabolism and gene expression as sit is a dynamic transcriptional mechanism highly responsive to external stimuli and environmental changes such as nutrient availability [37]. Thus, it is intriguing to hypothesize a role for TRAP1 network in the regulation of DNA remodeling because of its ability to reprogram cancer cell fate and metabolism. However, further studies are needed to investigate more deeply the mechanism of TRAP1 regulation of LRP5 expression and to prove its connection with TRAP1 regulation of bioenergetics.

In conclusions, this study suggests that Wnt/β-Catenin signaling is modulated at multiple levels by TRAP1 protein network and that the modulation of LRP5 and LRP6 receptors represents an additional mechanism responsible for TRAP1 regulation of stemness maintenance in human CRC.

## 4. Materials and Methods 

### 4.1. Cell Line and Patients-Derived Spheroids

Human CRC k-ras G13D HCT116 and embryonic kidney HEK293T cells were purchased from American Type Culture Collection (ATCC, Manassas, VA, USA). Cell lines were routinely monitored in our laboratory by microscopic morphology, whereas cell line authentication was verified before starting this study by STR profiling, according to ATCC product description. HEK293T cells were cultured in DMEM supplemented with 10% (*v/v*) FBS, 1.5 mM glutamine, and 100 U/mL penicillin–streptomycin, and HCT116 cells were cultured in McCoy’s medium supplemented with 10% (*v/v*) FBS, 0.75 mM glutamine, and 10 U/mL penicillin–streptomycin. Unless otherwise specified, reagents were purchased from Thermo Fisher Scientific (Waltham, MA, USA). ShTRAP1 HCT116 cells were cultured as previously reported [37].

CSCs derived from human CRCs were kindly provided by Professor Ruggiero De Maria (General Pathology Institute, Catholic University, Rome, Italy). Experiments were performed according to the Ethics Approval obtained by Prof. De Maria from the Ethics Committee of the Istituto Superiore di Sanità (Rome, Italy) and a Material Transfer Agreement was signed between the Legal Representatives of the two Research Institutes. CSCs were cultured as spheroids in ultra-low attachment tissue culture flasks (Corning Costar, Cambridge, MA, USA) in humidified atmosphere at 37 °C and 5% CO_2_ in Advanced DMEM F12 supplemented with 10 mM HEPES, 100 ng/mL human recombinant bFGF, 10 mM Nicotinamide, 2 mM L-Glutamine, 100 U/mL P/S, N-2 supplement 1X, B-27 supplement 1X, and 50 ng/mL human recombinant EGF. Spheroids were passaged weekly by mechanical dissociation or by incubation for 3–5 min at 37 °C with TrypLE Express. 

### 4.2. Transfection Procedures

TRAP1 transient silencing were performed with two independent siRNAs purchased from Qiagen (Milano, Italy; TRAP1 siRNA #1 cat. n. SI00115150 and TRAP1 siRNA #2 cat. n. SI00115164). TRAP1 siRNA #1 was used for the vast majority of the experiments, unless otherwise specified. TBP7 transient silencing were performed with siRNAs SI00301469 (Qiagen, Milano, Italy). For control experiments, cells were transfected with a similar amount of control siRNA (Qiagen, cat. n. SI03650318). Transient transfections of siRNAs were performed using HiPerFect Transfection Reagent (Qiagen) according to the manufacturer’s protocol. The Wnt pathway activation was evaluated by the Cignal TCF/LEF Reporter Assay Kit (GFP) (CCS-018G Qiagen) according to the manufacturer’s protocol. TRAP1 cDNA was transfected as previously reported [18]. 

### 4.3. Immunoblot Analysis 

Total cell lysates were obtained by homogenization of cell pellets and tissue samples in a cold lysis buffer (20 mM Tris, pH 7.5 containing 300 mM sucrose, 60 mM KCl, 15 mM NaCl, 5% (*v/v*) glycerol, 2 mM EDTA, 1% (*v/v*) Triton X-100, 1 mM PMSF, 2 mg/mL aprotinin, 2 mg/mL leupetin, and 0.2% (*w/v*) deoxycholate) for 30 min on ice. Immunoblot analysis was performed as previously reported [24]. Protein immunoprecipitation was carried out starting from 1 mg of total protein extracts by Pierce Classic IP kit (Thermo Scientific, Waltham, MA, USA) according to manufacturer’s protocol.

The following antibodies from Santa Cruz Biotechnology (Dallas, TX, USA) were used: mouse monoclonal anti-HSP75 (sc-73604), mouse monoclonal anti-PSMC4 (TBP7) (sc-166003), mouse polyclonal anti-MCT4 (sc-50329), mouse monoclonal anti-Ubiquitin (sc-8017), mouse monoclonal anti-PKM2 (sc-365684), mouse monoclonal anti-LDH (sc-137243), rabbit polyclonal anti-SOX2 (sc-20088), mouse monoclonal anti-c-Myc (sc-40), mouse monoclonal anti-GAPDH (sc-47724), mouse monoclonal anti-Tubulin (sc-8035) and mouse monoclonal anti-β-Actin (sc-47778). The following antibodies from Cell Signaling Technology (Boston, MA, USA) were used: rabbit monoclonal anti-active-β-Catenin (#8814), rabbit polyclonal anti-total-β-Catenin (#9562), rabbit monoclonal anti-LRP6 (#3395), rabbit polyclonal anti-phosphoLRP6 (#2568), rabbit monoclonal anti-LRP5 (#5731), rabbit monoclonal anti-TCF1 (#5731), and rabbit monoclonal anti-Survivin (#2808). The following antibody was also used; rabbit monoclonal anti-Axin2 (ab109307, Abcam).

Protein levels were quantified by densitometric analysis using the Quantity One 4.5 software (BioRadLaboratories GmbH, Hercules, CA, USA) and normalized according to the expression of the housekeeping gene.

### 4.4. RNA Extraction and Real-Time RT-PCR

Total RNA was extracted from cell pellets and CRC specimens using the TRIzol reagent (Life Technologies, Carsbad, CA, USA). RNA concentrations were determined spectrophotometrically at 260 using the NanoDrop™ 2000/2000c Spectrophotometer (Thermo Scientific). Each RNA was transcribed to cDNA using a Transcriptor First Strand cDNA Synthesis Kit (Roche, Mannheim, Germany) according to the manufacturer’s instructions. For real-time PCR analysis, 0.5 ng of cDNA samples was amplified using the LightCycler 480 SYBR Green I Master (Roche) in a LightCycler 480 (Roche) according to the manufacturer’s instructions. The following primers were used; TRAP1 forward 5′-CGCAGCATCTTCTACGTGC-3′, reverse 5′-CTGATGAGTGCGCTCTCC-3′ (PCR product 200 bp); LRP6 forward 5′-ACAGAGCTGCAATGGATG-3′, reverse 5′-TGAGCCCAAGCATATTTG-3′ (PCR product 150 bp); LRP5 forward 5′-AGACCAATAACAACGACGTG-3′, reverse 5′-TTCTTGCCCATCCAGTC-3′ (PCR product 211 bp); β-Actin forward 5′-CGCAAAGACCTGTACGC-3′, reverse 5′-CACACGGAGTACTTGCGC-3′ (PCR product 152 bp). Reaction conditions were as follows; pre-incubation at 95 °C for 5 min, followed by 45 cycles of 10 s at 95 °C, 10 s at 59 °C, and 10 s at 72 °C. β-Actin was chosen as an internal control.

### 4.5. Methylation Specific PCR (MS-PCR)

Genomic DNA (gDNA) from cell pellets and CRC specimens was extracted by using the QIAamp DNA Mini kit (Qiagen). DNA concentration and purity were assessed by NanoDrop technology (Thermo Scientific) using, respectively, the absorbance at 260 nm and the 260/280 nm absorbance ratio. Five-hundred nanograms of total gDNA was treated with sodium bisulfite using the Zymo EZ DNA Methylation Kit (Zymo Research D5002), according to the manufacturer’s protocol. The presence of methylation in bisulfite converted gDNA was detected by MS-PCR [38,39]. Primer sequence used for PCR reaction are LRP5 right methylated 5′-AAATCTACTTAATAACCTCCTCGCT-3′, LRP5 left methylated 5′-GTTTTTGTTATTTGTTAATCGTCG-3′, LRP5 right unmethylated 5′-ATAAATCTACTTAATAACCTCCTCACT-3′, LRP5 left unmethylated 5′-GTTTTTGTTATTTGTTAATTGTTGG-3′. PCR reaction conditions were as follows: pre-incubation at 95 °C for 10 min, followed by 35 cycles of 30 s at 95 °C, 60 s at 59 °C (primers specific for methylated LRP5) or 56.7 °C (primers specific for un-methylated LRP5), 60 s at 72 °C.

### 4.6. Flow Cytometry 

Cell suspensions were reconstituted to a final concentration of 1.0 × 10^5^ cells/mL in PBS containing 0.1% (*w/v*) NaN_3_ and 5% (*v/v*) FBS. As a preliminary step, intrinsic cell fluorescence was analyzed, using unlabeled samples. For specific staining, 100 μL of each cell suspension were incubated with 10 μL of mouse monoclonal anti-CD44 (APC) (559942) from Becton Dickinson (San Jose, CA, USA) for 15 min at room temperature. Cells were finally analyzed by flow cytometry with Navios (Beckman Coulter, Brea, CA, USA) and data were processed by Kaluza 2.1 software. 

### 4.7. Sphere-Forming Assay 

Cells (1.25 × 10^4^) were suspended in a medium containing Advanced DMEM F12 supplemented as described above, seeded in ultra-low attachment tissue culture flasks, and left growing for 3 days in humidified atmosphere at 37 °C and 5% CO_2_. Density of transformation colonies were compared by cell counts and represented as percentage of colonies in TRAP1-silencing conditions respect to the respective siNeg control (means ± S.D).

### 4.8. Tumor Specimens and TCGA Analysis

Specimens from 41 human CRCs and corresponding normal, non-infiltrated, peritumoral mucosa were obtained from the IRCCS-CROB Tissue Biobank. Patients’ baseline characteristics are reported in Table 1. Express written informed consent to use biologic specimens for investigational procedures was obtained from all patients. In order to compare levels of TRAP1, active β-Catenin, LRP5, and LRP6 in tumor specimens, protein levels were quantified by densitometric analysis using the Quantity One 4.5.0 software (BioRad Laboratories GmbH, Hercules, CA, USA) and expressed as time increase/decrease in tumors compared with the respective peritumoral non-infiltrated mucosa (Table S1). 

Expression and methylation data of TCGA samples were downloaded from XENA data repository [40]. To evaluate the magnitude and significance of the association between LRP5 mRNA expression and promoter methylation in TCGA samples we calculated the Spearman’s rank correlation rho and performed a correlation test. The statistical analysis was performed in R (R version 3.6.1. Vienna, Austria) [41].

### 4.9. Statistical Analysis 

The two-sided paired t-test was used to establish the statistical significance between different variable in silenced/transfected/treated cells and related controls. The Spearman test was used to establish the statistical significance of the correlation between TRAP1, β-Catenin, LRP5, and LRP6 levels in human CRCs. Statistically significant values are reported in figure legends. All experiments were independently performed at least three times and three technical replicates were used for statistical analysis. Data represent means + S.D.

## Figures and Tables

**Table 1:** Baseline characteristics of the patients.

Patients	41	
Age (years)		
Median	69	
Range	36–89	
Sex	n°	
Female	16	
Male	25	
Tumor stage	n°	(%)
T1	2	5
T2	3	7
T3	30	73
T4	6	15
N0	18	44
N1	14	34
N2	9	22
M0	32	78
M1	9	22

## Supplementary Materials

Supplementary materials can be found at https://www.mdpi.com/1422-0067/21/20/7526/s1. Figure S1: Statistical analysis of densitometric data from three independent technical replicates of immunoblots reported in Figure 1B,D. Statistical significance respect to the respective siNeg control: * *p* < 0.05; ** *p* < 0.01; *** *p* < 0.001; n/s, not significant, Figure S2: Statistical analysis of densitometric data from three independent technical replicates of immunoblots reported in Figure 3A,B. Statistical significance respect to the respective siNeg control: * *p* < 0.05; ** *p* < 0.01; *** *p* < 0.001; n/s, not significant, Figure S3: Specificity of TRAP1 regulation of LRP5/6 receptors. (**A**) Total cell lysates from HCT116 cells silenced with control siRNA (siNeg) or two independent TRAP1 siRNAs (siTRAP1 #1 and #2) were separated by SDS-PAGE and immunoblotted with the indicated antibodies. (**B**) Total cell lysates from control (shNeg) or stable TRAP1-silenced (shTRAP1) HCT116 cells transfected with TRAP1 cDNA (pTRAP1) or pMOCK were separated by SDS-PAGE and immunoblotted with the indicated antibodies.

## Abbreviations

APC	Allophycocyanin
CRC	colorectal carcinoma
CSC	cancer stem cell
FBS	fetal bovine serum
FZD	Frizzled
LRP	low-density lipoprotein receptor-related protein
MFI	mean fluorescence intensity
PBS	phosphate-buffered saline
PE	Phycoerythrin
TRAP1	TNF receptor-associated protein 1
