# Supplementary material for: TRAP1 Regulates Wnt/β-Catenin Pathway through LRP5/6 Receptors Expression Modulation
Source: Int J Mol Sci. 2020 Oct 13;21(20):7526. doi: 10.3390/ijms21207526 (PMC7589514; doi:10.3390/ijms21207526)
Supplement: Supplementary file 1 [file ijms-21-07526-s001.pdf]

Supplementary Figure 1. Statistical analysis of densitometric data from three independent technical replicates of immunoblots reported in Figure 1B and D. Statistical significance respect to the respective siNeg control: *p<0.05; **p<0.01; ***p<0.001; n/s, not significant.

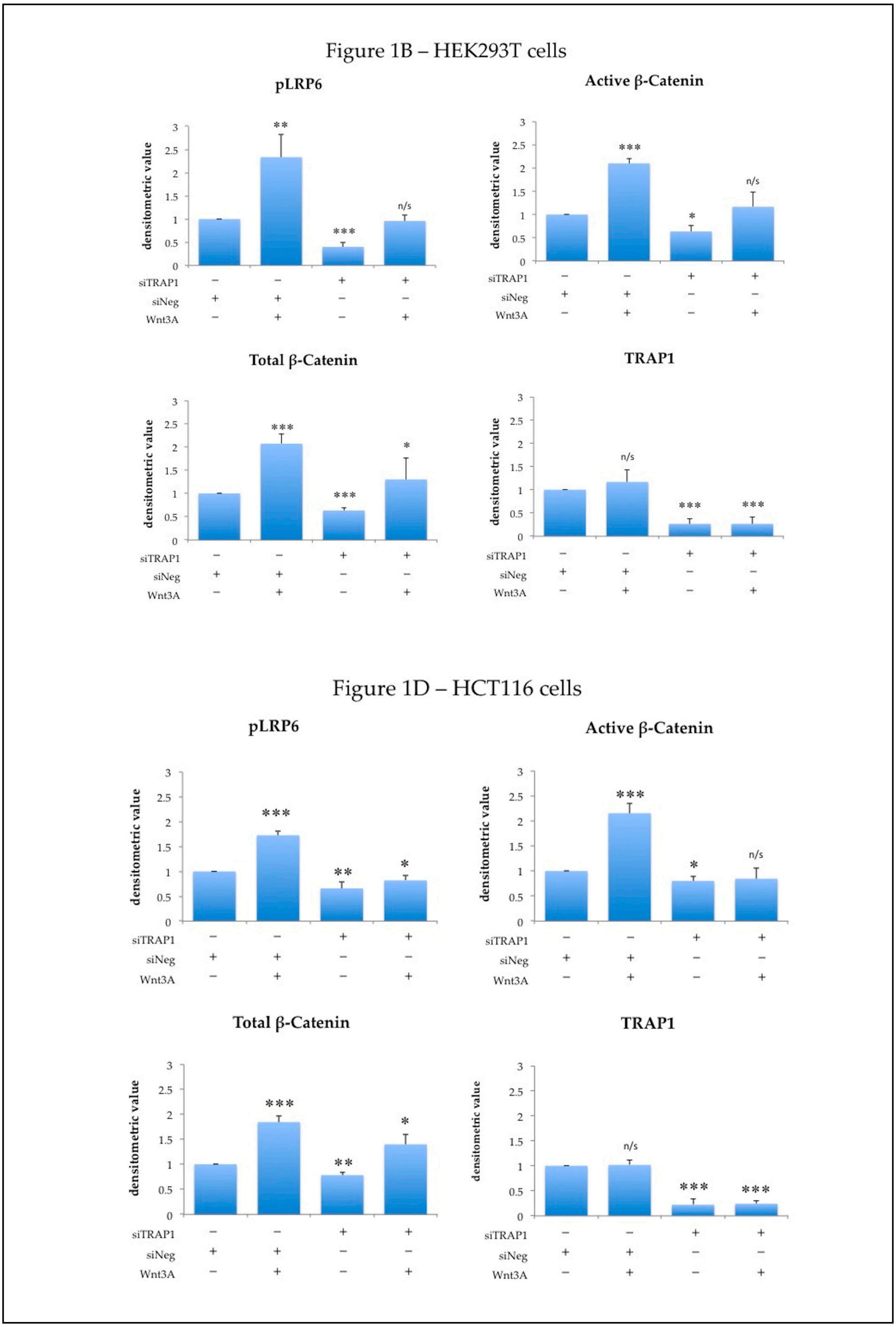

Supplementary Figure 2. Statistical analysis of densitometric data from three independent technical replicates of immunoblots reported in Figure 3A and B. Statistical significance respect to the respective siNeg control: *p<0.05; **p<0.01; ***p<0.001; n/s, not significant.

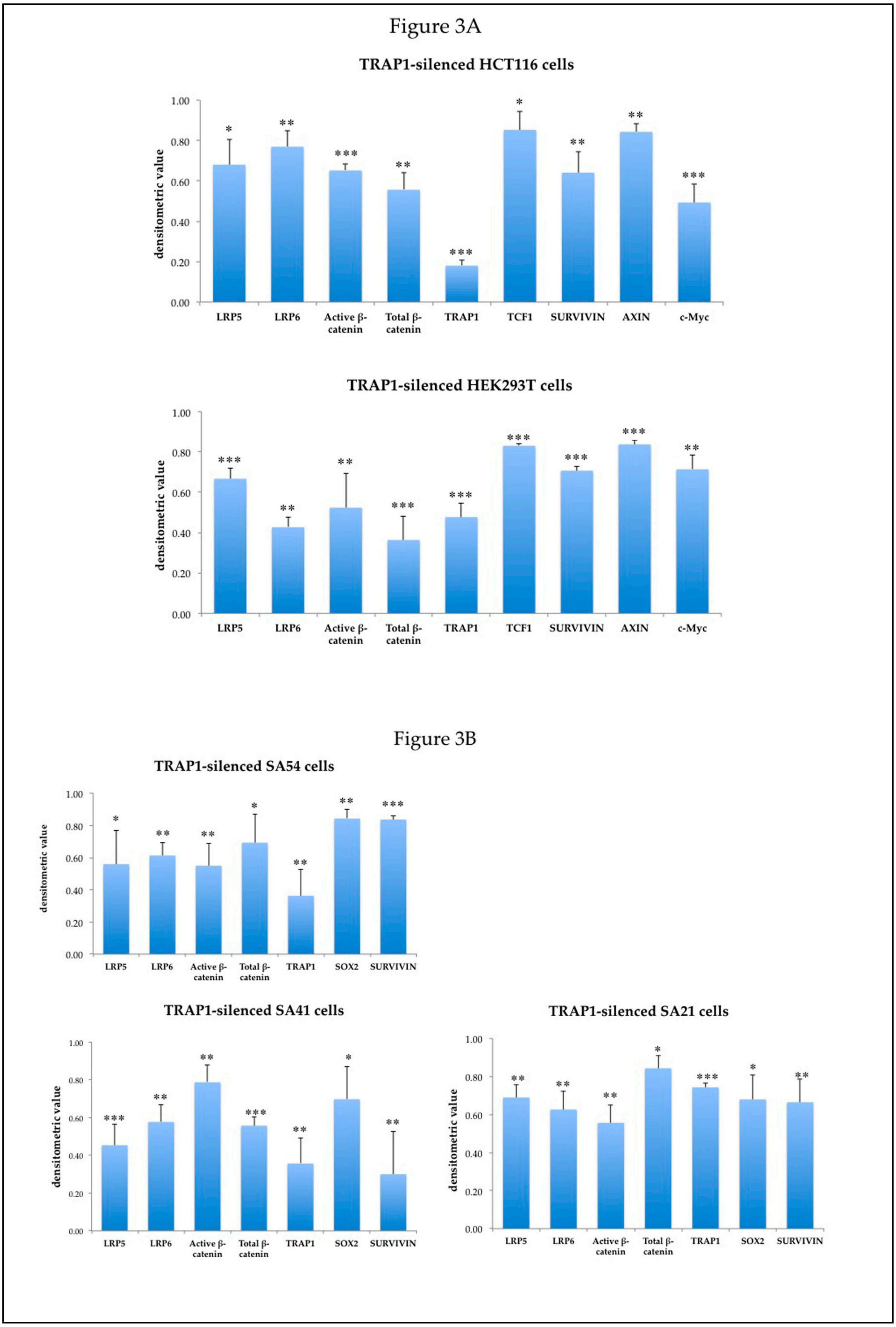

Supplementary Figure 3. Specificity of TRAP1 regulation of LRP5/6 receptors. (**A**) Total cell lysates from HCT116 cells silenced with control siRNA (siNeg) or two independent TRAP1 siRNAs (siTRAP1 #1 and #2) were separated by SDS-PAGE and immunoblotted with the indicated antibodies. (**B**) Total cell lysates from control (shNeg) or stable TRAP1-silenced (shTRAP1) HCT116 cells transfected with TRAP1 cDNA (pTRAP1) or pMOCK were separated by SDS-PAGE and immunoblotted with the indicated antibodies.

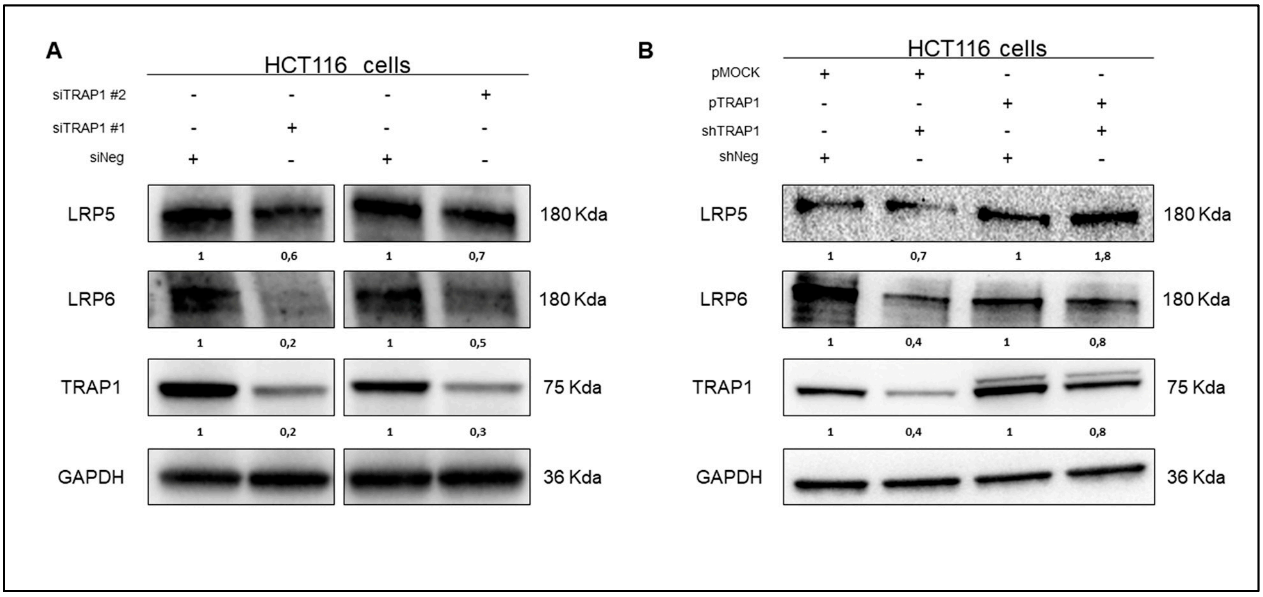

Supplementary Table S1. TRAP1, Active β-Catenin, LRP5 and LRP6 protein levels in human colorectal carcinomas expressed as time increase in tumor compared to correspondent non-infiltrated peritumoral mucosas.

Case N°	TRAP1	Active β-Catenin	LRP5	LRP6
1.	5.55	20.75	6.92	2.84
2.	0.08	0.11	0.00	0.67
3.	4.25	3.43	3.50	0.54
4.	12.26	13.53	15.79	0.38
5.	0.80	0.70	0.98	0.62
6.	0.03	0.01	0.2	0.91
7.	5.78	0.00	0.00	0.14
8.	0.70	0.12	0.29	0.44
9.	6.49	0.03	0.41	0.61
10.	1.02	0.00	0.05	0.89
11.	6.77	0.07	2.31	1.83
12.	6.27	0.00	3.19	0.90
13.	22.18	16.51	12.81	15.63
14.	4.71	3.14	0.44	0.75
15.	19.04	18.94	6.55	3.11
16.	4.05	0.41	0.73	0.95
17.	2.18	0.12	0.76	0.39
18.	12.02	2.10	0.36	0.78
19.	3.20	0.21	0.78	1.03
20.	0.69	0.65	0.52	1.04
21.	3.25	0.00	1.85	0.60
22.	7.80	0.12	3.76	1.55
23.	3.10	0.22	0.00	2.24
24.	8.07	2.08	3.05	2.65
25.	8.07	13.35	2.83	0.71
26.	5.03	0.29	1.84	0.99
27.	0.70	0.00	0.00	0.30
28.	2.85	0.76	0.89	0.83
29.	7.05	10.46	1.55	0.42
30.	0.00	0.00	0.00	0.04
31.	0.12	0.09	0.00	0.00
32.	12.85	0.64	0.65	0.65
33.	4.95	0.00	2.73	0.81
34.	1.15	0.14	0.07	0.37
35.	12.97	0.31	1.87	1.56
36.	6.28	5.38	14.11	9.23
37.	0.86	1.80	0.18	0.27
38.	8.97	9.73	6.46	1.95
39.	7.27	2.50	2.04	2.15
40.	1.12	12.80	2.12	2.04
41.	4.71	1.65	6.35	2.65
